# Cardiovascular Disease in Primary Sjögren’s Syndrome: Raising Clinicians’ Awareness

**DOI:** 10.3389/fimmu.2022.865373

**Published:** 2022-06-09

**Authors:** Mihnea Casian, Ciprian Jurcut, Alina Dima, Ancuta Mihai, Silviu Stanciu, Ruxandra Jurcut

**Affiliations:** ^1^ Cardiology Department, University of Medicine and Pharmacy "Carol Davila", Bucharest, Romania; ^2^ 2^nd^ Internal Medicine Department, Central Military University Emergency Hospital, Bucharest, Romania; ^3^ Department of Rheumatology, Colentina Clinical Hospital, Bucharest, Romania; ^4^ Rheumatology Department, University of Medicine and Pharmacy "Carol Davila", Bucharest, Romania; ^5^ Cardiac Noninvasive Laboratory, Central Military University Emergency Hospital, Bucharest, Romania; ^6^ Internal Medicine Department, University of Medicine and Pharmacy "Carol Davila", Bucharest, Romania; ^7^ Department of Cardiology, Expert Center for Rare Genetic Cardiovascular Diseases, Emergency Institute for Cardiovascular Diseases, Bucharest, Romania

**Keywords:** Sjogren’ syndrome (SS), cardiovascular risk (CV risk), strain echocardiography, inflammation, atherosclerosis, autoimmune disease (AD), cardiac magnetic resonance imaging (CMR)

## Abstract

In the ever evolving landscape of systemic immune mediated diseases, an increased awareness regarding the associated cardiovascular system impairment has been noted in recent years. Even though primary Sjögren’s Syndrome (pSS) is one of the most frequent autoimmune diseases affecting middle-aged individuals, the cardiovascular profile of this specific population is far less studied, at least compared to other autoimmune diseases. Traditional cardiovascular risk factors and disease specific risk factors are inextricably intertwined in this particular case. Therefore, the cardiovascular risk profile in pSS is a multifaceted issue, sometimes difficult to assess. Furthermore, in the era of multimodality imaging, the diagnosis of subclinical myocardial and vascular damage is possible, with recent data pointing that the prevalence of such involvement is higher in pSS than in the general population. Nevertheless, when approaching patients with pSS in terms of cardiovascular diseases, clinicians are often faced with the difficult task of translating data from the literature into their everyday practice. The present review aims to synthesize the existing evidence on pSS associated cardiovascular changes in a clinically relevant manner.

## 1 Introduction

Primary Sjogren’s syndrome (SS) is one of the most frequent autoimmune diseases, with variable prevalence rates (between 13.1 and 60.8 per 100.000 inhabitants), depending on the classification criteria used and the geographical areas in question (1–3). As with other autoimmune diseases, it features a strong female propensity, affecting middle-aged Caucasian women, with a female/male ratio between 6 and 10.7 (1–3). In SS the secretory activity of the exocrine glands (lacrimal and/or salivary glands) is disrupted by chronic progressive lymphocyte infiltration (4). Even though the exocrine glands are the most affected sites, the deleterious effects of the immune dysregulation extend far beyond them having an important systemic impact, as in the case of other classical autoimmune diseases (e.g. rheumatoid arthritis - RA, scleroderma – SSc, systemic lupus erythematous - SLE) (5). Therefore, in SS the classical clinical features of xerophthalmia and xerostomia are accompanied by systemic involvement, such as skin, joints, muscles, peripheral and central nervous system, kidneys, lungs or liver and laboratory abnormalities (particularly hypergammaglobulinemia and hypocomplementemia) which plays an important role in the general prognosis of the disease (6, 7). Among them, the cardiovascular manifestations, lymphoid malignancies, or associated infections are leading causes of morbi-mortality in SS (8, 9).

The true prevalence of extraglandular manifestations (EGM) in patients with pSS remains difficult to estimate, with some limited data suggesting that the mean prevalence rates of EGM is 42% (10). The great variability seen in the literature for EGM rates can be explained by the application of different methods for diagnosing organ damage, underdiagnosis of early and/or subclinical organ damage and lack of scrutiny. In addition, while some EGM are included in the main disease activity indexes (Sjogren’s Syndrome Disease Damage Index- *SSDAI* and EULAR Sjogren’s Syndrome Disease Activity Index- *ESSDAI*), others are not, hence, their prevalence may be underreported (10). One study reported that 20% of the patients with primary SS developed EGM not featured in the disease activity indexes (11). Cardiovascular manifestations are the most frequent organ-specific group of non-ESSDAI features (11).

The present review aims to synthesize the existing evidence on SS associated cardiovascular changes in a clinically relevant manner.

## 2 Cardiovascular Risk in Sjogren’s Syndrome

**Figure 1:**
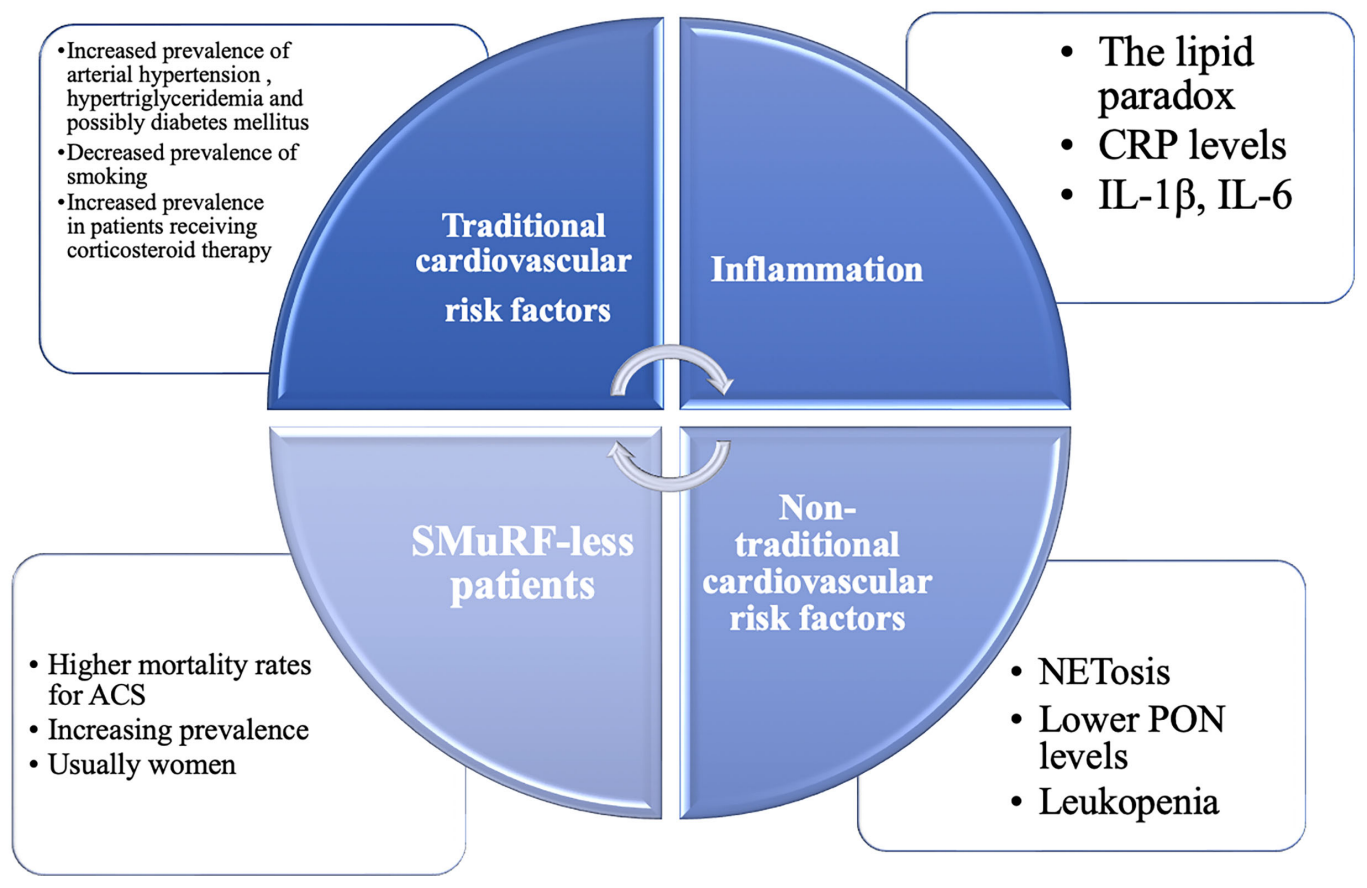
The continuum of cardiovascular risk factors in primary Sjögren’s Syndrome (pSS). Traditional and non-traditional cardiovascular risk factors are inextricably intertwined in this heterogenous population. CRP, C reactive protein; GC, glucocorticoids; HCQ, hydroxychloroquine; IL, interleukin; NETosis, formation of neutrophil extracellular traps; NSAIDs, non-steroidal anti-inflammatory drugs; PON, paraoxonase-1; SMuRF-less, without standard modifiable cardiovascular risk factors.

Patients with pSS represent an interesting population in terms of cardiovascular risk factors, displaying both traditional risk factors (seen in the general population) and specific, non-traditional, risk factors (see **Figure 1**). Being a heterogenous disease, stratification is essential when considering such a broad subject. A recent computational analysis revealed that there are two distinct patterns in the distribution of cardiovascular events among SS patients (12–14). In the first pattern, there is a close interconnection between traditional risk factors and glandular involvement, while in the second pattern, extra-glandular disease activity (purpura, leukopenia, hypocomplementemia, cryoglobulinemia) and longer disease duration are associated with cardiovascular events (12). It has been established by several studies and a meta-analysis that pSS is associated with an increased risk of major adverse cardiovascular events (MACEs), such as cerebrovascular events (RR = 1.46 [95% CI 1.43- 1.49]; P < 0.00001) and coronary events (RR = 1.34 [95% CI 1.06-1.38]; P = 0.001), with disease-related clinical and immunological markers playing a role in promoting CV events, in addition to traditional cardiovascular risk factors (15, 16). Another meta-analysis also supports the finding that patients with pSS have a higher risk of cardiovascular disease (OR = 1.30 [95% CI 1.09-1.55]; P = 0.03), while there was no significant difference concerning the risk of cerebrovascular events (OR=1.31 [95% CI 0.96-1.79]; P = 0.09) (17). However, as opposed to RA or SLE, the risk of cardiovascular mortality does not appear to be higher in pSS compared to the general population (16). Therefore, in terms of cardiovascular risk factors, MACEs and cardiovascular mortality, pSS patients represent a particular case among patients with other autoimmune diseases. The clinically relevant key messages of this chapter are summarized in **Table 1**.

**Table 1:** Clinically relevant key messages based on topics discussed.

Topic	Key messages
Cardiovascular risk factors	Some traditional cardiovascular risk factors are more prevalent in patients with pSS than the general population. A patient centered approach should be considered Correction of traditional risk factors is warranted Control of systemic inflammation
Structural myocardial disease	General screening is not recommended Subclinical myocardial changes can be diagnosed using advanced echocardiographic techniques (e.g. speckle-tracking echocardiography) or CMR Search for in symptomatic patients or in the context of ECG abnormalities
Venous thrombosis	The risk for venous thrombosis is higher in SS patients than in the general population, although the risk is not evenly distributed among SS patients.
Pulmonary hypertension	Multiple mechanisms possible General screening is not recommended Screening in patients with RP could be useful
ECG abnormalities	ECG at baseline and during follow-up Aim to avoid situations leading to LQTS Holter monitoring should be considered in symptomatic patients with LQTS
Aortic disease	Screening according to symptoms
Autonomic abnormalities	Screening according to symptoms

### 2.1 Traditional Cardiovascular Risk Factors

Patients with pSS are a heterogeneous population in terms of age and comorbidities. Therefore, assessing the true prevalence of traditional risk factors is difficult, with conflicting data emerging from different cohorts. Traditional risk factors, such as hypertension, hypertriglyceridaemia and metabolic syndrome appear to be more prevalent in some patients with pSS (a twofold higher prevalence), whereas smoking, obesity and diabetes are less prevalent (15, 18, 19). In other studes, patients exhibited a twofold higher prevalence of diabetes mellitus (20–22). Diabetes mellitus appeared to be more prevalent in cohorts of Spanish patients, highlighting the importance of genetic and metabolic brackground (20, 22, 23). Regardless of the incongruous data from the literature, one should keep in mind that the association between pSS and diabetes mellitus has been demonstrated by several studies, confirming the common autoimmune background between the two diseases (23). Interestingly, the presence of cardiovascular risk factors was associated with a higher prevalence of EGM, raised CRP levels and a lower frequency of hypergammaglobulinaemia and anti-Ro antibodies (20). It is unclear whether the expression of these risk factors in patients with pSS, particularly hypertension and dyslipidemia, is in relation with the disease duration, disease activity or treatment. It is well known that corticosteroid therapy is associated with a higher prevalence of cardiovascular risk factors, particularly diabetes mellitus, hypertension and hypertriglyceridaemia in patients with pSS (20). Therefore, as with other rheumatological diseases, despite a high cardiovascular disease and risk factors burden, the assessment and management of traditional and modifiable cardiovascular risk factors remains inadequate (24). As the majority of patients with pSS are women, this could be partially explained by the fact that women are underrepresented in cardiovascular studies and cardiovascular diseases are underdiagnosed and undertreated in women (25). Equally, cardiovascular risk factors are underappreciated in women, leading to a poor management of modifiable cardiovascular risk factors (26). In the general population, several risk scores are validated and used to accurately assess the cardiovascular risk of individuals of developing cardiovascular diseases, MACEs or their cardiovascular mortality rates. Individuals with autoimmune diseases, notably RA, have a higher cardiovascular risk than their peers (27). In individuals with RA, the Systemic Coronary Risk Estimation (SCORE) should be multiplied by 1.5 to adequately assess the cardiovascular risk (27). However, pSS per se is not recognized as a specific clinical condition to prompt risk reclassification in any of the risk scores. Available data highlights that patients with pSS have a significantly higher degree of subclinical atherosclerosis, with increased arterial wall thickening and higher pulse wave velocities compared to healthy controls, even if their Framingham Risk Scores were similar (28, 29). The use of cardiovascular prediction tools as for the general population is currently recommended (30). Accordingly, the question remains if the cardiovascular risk, as calculated by different scores, matches the real cardiovascular risk of patients with pSS and if so, how reliable they are in predicting MACEs and cardiovascular mortality in this specific population.

### 2.2 Specific Risk Factors and Possible Mechanisms

There has been an increasing awareness of the importance of non-traditional and immune factors in cardiovascular diseases. Recently, a new risk group has emerged: patients without standard modifiable cardiovascular risk factors (SMuRF-less) but higher than average cardiovascular risk (31). Interestingly, even though they are overlooked, the proportion of SMuRF-less patients is increasing, while their in-hospital mortality rates when experiencing an acute coronary syndrome for instance, are higher than their peers with at least one SMuRF, and this appeared particularly evident in women (31). These findings are crucial in shaping the real cardiovascular risk of patients with pSS, as they also tend to associate non-traditional, underacknowledged risk factors. Immunological, thrombotic and pro-atherogenic mechanisms, antibody mediated endothelial dysfunction, neutrophil cellular activation and pro-inflammatory cytokines are present in patients with pSS, even though their contribution to the cardiovascular risk is unknown, underestimated and far from being understood. Accordingly, the lipid paradox has been described in autoimmune diseases (32, 33). The lipid paradox marks the inverse relationship between low levels of LDL and increased risk of cardiovascular diseases, in the setting of active inflammation (32, 33). Conversely, despite the increase in LDL levels, vascular risk surrogates are favourably modified when the inflammatory burden is reduced with biological therapy, possibly explained by alteration in the composition of HDL particles (34).

Neutrophil extracellular traps (NETs) are a regulated form of neutrophil cellular death, first described in 2004 (35). Formation of NETs is known as NETosis. Since its discovery, studies demonstrated the role of NETosis in the development of autoimmune diseases, such as SLE, RA and pSS (36, 37). Even more, NETosis might contribute to atherosclerosis progression and is also a pathogenic factor for heart failure, although these findings are not specific for patients with pSS (38, 39). In the light of these findings, NETosis is a common pathway in the progression of pSS and cardiovascular diseases and could be one of the keys in explaining the cardiovascular profile of patients with pSS and other autoimmune diseases.

Hematological abnormalities are common in patients with pSS, with leukopenia and lymphopenia being considered markers of disease activity. Leukopenia also appears to be a marker for vascular damage in pSS, being associated with macrovascular impairment of endothelium-independent function and intima-media thickening (40). Not surprisingly, patients with leukopenia exhibited a sixfold higher risk of developing angina, even though they tended to lack traditional cardiovascular risk factors (15).

Oxidative stress is an emerging non-traditional risk factor for cardiovascular diseases. Recent attempts have been made to quantify it by measuring levels of paraoxonase-1 (PON), which protects LDL particles from oxidation (41). It appears that PON levels are lower in patients with SS, regardless of steroid intake, which generally affects the circulating levels of LDL particles (41).

In pSS, the abnormal activation of B and T lymphocites will lead to an increased production of various cytokines, such as interleukin (IL)–1β and IL-6, perpetuating the inflammatory response (42). Increased circulating levels of IL-1β, IL-6 and C- reactive protein (CRP) also promote atherosclerosis, independently of circulating levels of LDL particles (43) (44),. IL-1β is of particular interest in understanding the cardiovascular risk of patients with pSS, as its levels were found to be higher in pSS patients with metabolic syndrome (19).

### 2.3 Treatment and the Cardiovascular Risk

Traditional and specific cardiovascular risk factors are intertwined in patients with pSS. In addition, as pSS is a chronic systemic disease, patients are usually exposed to different therapies, for short and/or long periods of time, with an impact on their comorbidities and cardiovascular risk as previously mentioned. The significant cardiovascular protective effect of hydroxychloroquine (HCQ) therapy was reported (45). The prevalence of cardiovascular risk factors in patients with pSS was lower in those treated with hydroxychloroquine (46). Unsurprisingly, the use of HCQ was associated with a lower risk of death and lower incidence of coronary artery disease among patients with pSS, with some potential benefits in modulating the endothelial dysfunction and the pro-inflammatory cytokines (45).

Non-steroidal anti-inflammatory drugs (NSAIDs) are generally associated with an increased risk of MACE in the general population, while their use did not lead to a significant increase in the risk of MACE in pSS patients (45). Furthermore, while higher doses of glucocorticoids (GC) are associated with dyslipidemia, diabetes mellitus and coronary artery disease, because lower doses of GC are used in SS and for shorter periods of time, their impact on the risk of cardiovascular events seems to be unsignificant (45). The use of immunosuppressive therapy was associated with a higher risk of cardiovascular events, but its real impact in patients with SS needs further studies (15). On the other hand, the role of biological therapy in reducing the rate of MACEs and the cardiovascular risk, independently of lipid-level lowering, in the general population, as well as in patients with autoimmune diseases, is emerging, as shown by several studies (32, 44, 47). Even if the beneficial effect of different biological therapies on subclinical atherosclerosis has been observed in RA, the impact in pSS is unclear, as currently no disease-modifying drug has been approved in pSS and trials with biological therapies have been completed with mixed results (32, 48).

## 3 Cardiovascular Involvement in Sjogren’s Syndrome

### 3.1 The Pathophysiology of Vascular Disease in SS

Subclinical vascular disease has been reported in autoimmune diseases, with inflammation playing a pivotal role in generating endothelial dysfunction and arterial stiffness leading to structural changes and accelerating atherosclerosis (49, 50). Endothelial dysfunction is actually regarded as one of the first steps in the development of subclinical atherosclerosis and patients with pSS exhibit endothelial injury and abnormal endothelial function restoration (51). It is still debatable which parameter of disease (activity or duration) has a greater impact on the development of subclinical atherosclerosis. It appears that disease duration reflecting a longer period of systemic inflammation seems rather critical, at least in the case of other autoimmune diseases (52). The clinically relevant key messages of this chapter are summarized in **Table 1**.

#### 3.1.1 The Spectrum of Subclinical Vascular Manifestations in SS

Arterial stiffness (AS) reflects the mechanical tension and elasticity of the large caliber blood vessels. It is a known independent predictor for vascular related morbidity and mortality, being one of the earliest detectable manifestations of adverse structural and functional changes within the vessel wall, reflecting the cumulative effect of cardiovascular risk factors on vascular aging (53, 54). Hence, might be an useful clinical tool for risk stratification (54). The main parameter used to assess AS is the pulse wave velocity (PWV), as estimated by noninvasive methods, such as the carotid-femoral PWV. An indirect measure of AS can be achieved by using the augmentation index (AI), which measures the augmentation of central aortic pressure by a reflected pulse wave (55). CV risk assessment in chronic inflammatory and autoimmune diseases should also rely on PWV and AI, however this recommendation was not formulated based on studies including SS patients (52). AS is increased in chronic inflammatory and autoimmune diseases (52). In a metanalysis using data from 8 different observational studies involving 767 subjects, a significant increase in PWV was observed in patients who have pSS compared with controls (17). The increased AS associated with pSS can be in the context of traditional cardiovascular risk factors (age, blood pressure and LDL levels) or caused by the use of steroids and their secondary side effects (dyslipidemia, hypertension), being unclear if the disease itself is responsible for this particular finding (53). In addition, no relationship has been established so far between disease activity and AS (52).

AS should also be regarded as the link between the vascular and myocardial disease, since increased AS leads to diastolic dysfunction and consequently heart failure with preserved ejection fraction (52). Its relationship with diastolic dysfunction has been investigated in one study which involved 50 SS patients. As expected, aortic distensibility had significant correlations with E/A ratio and isovolumetric contraction time. Therefore, aortic elasticity parameters can be used to predict not only vascular involvement, but also subclinical cardiac involvement in SS patients (56).

Regarding structural vascular changes, patients with primary SS also have higher IMT compared to healthy subjects, suggesting that pSS is associated with subclinical atherosclerosis (17, 29).

#### 3.1.2 Raynaud Phenomenon

Raynaud phenomenon (RP) is characterized by an abnormal vascular response to cold and emotional stress originating in the peripheral arterial circulation. Its main features are recurrent, reversible spasms, resulting in the clinical triad of ischemia and cyanosis, followed by hyperemia. With regard to pSS, RP is a common vascular feature, found in up to 20% of the patients (11, 57). As with systemic sclerosis (SSc), RP might be one of the earliest signs of the disease, appearing even before the sicca symptoms (57). However, in the case of pSS, RP is milder, with less severe vascular complications and less need for pharmacological interventions compared to SSc (57). SLE patients with RP were found to associate an elevated systolic pulmonary arterial pressure value and recent data indicate that RP is a significant risk factor for developing pulmonary arterial hypertension in pSS as well (OR 9.660, p= 0.000) (58) ^(^ 59 ^),^. Additionally, it appears that RP, primary or due to connective tissue diseases, is associated with a reduction in myocardial perfusion reserve index (MPRI), assessed by stress perfusion cardiac magnetic resonance (60). Interestingly, in the case of secondary RP, the reduction in MPRI was more severe than in the case of primary RP (0.7 ± 0.2 vs. 1.7 ± 0.6, P < 0.001), possibly in the context of occult myocardial fibrosis (60). Furthermore, RP is a high risk factor for left-ventricular regional dysfunction in SSc (61). A capillaroscopic study and determination of serum anticentromere and topoisomerase I antibodies are currently recommend in SS patients with RP, as well as a closer surveillance since some patients might actually develop overt SSc during follow-up (57). In these cases, the SS might be considered secondary to SSc, with sicca symptoms appearing well before other clinical manifestations. Even in the absence of suggestive clinical features, patients with SS and RP should be screened for the presence of cryoglobulins, one of the classical possible associations in these patients with important prognostic implications.

#### 3.1.3 Aortic Disease

Apart from the increased prevalence of traditional cardiovascular risk factors (hypertension notably), increased arterial stiffness and accelerated atherosclerosis previously discussed, the molecular pathways responsible for the destruction of salivary glands in pSS, such as MAPK, TGF-ß and MMP, can also affect the aortic matrix (62). A greater incidence of aortic aneurysms (AA) and aortic dissections (AD) has been observed in patients with pSS in a nationwide population-based cohort study (0.43% vs. 0.37%, P = 0.045) (62). The study highlighted that patients with pSS have an increased risk of AA or AD (AHR = 1.753, P = 0.042), compared to the general population, while interestingly, the risk seems to be even higher in patients with secondary SS (AHR = 3.693, P < 0.001) (62). Although no cause-effect relationship has been formally established, other studies identified an independent association between AA and RA (OR = 1. 406, 95%; CI 1.094-1.789, P = 0.006) and also AA and SLE (OR = 20.6, 95%; CI 1.21-3.51, P < 0.01) (63, 64). For now, even if the screening for AD or AA is not currently recommended in patients with SS and should be individualized (according to clinical manifestations, disease length or other imaging features), clinicians should be aware these complications are more frequent in patients with pSS.

#### 3.1.4 Venous Thrombosis

If we consider Virchow’s triad of stasis, endothelial injury and hypercoagulability, SS might be regarded as a risk factor for venous thrombosis occurrence (VT). This has already been established by different studies which concluded that patients with primary SS have a substantially increased risk of VT, which is even 7 times higher, and that the risk seems to be higher in the first year after diagnosis, when the disease is usually least controlled (65–68). However, as pSS is a heterogenous clinical entity, including different subpopulations, not all patients carry the same risk for VT, as seen in a metaanalysis in which SS patients showed the widest confidence intervals for VTE incidence compared to other autoimmune diseases (68). The spectrum of clinical manifestations associated with venous thrombosis in pSS comprises different entities, ranging from the typical sites, such as deep vein thrombosis (DVT) and pulmonary embolism (PE) and atypical sites, such as cerebral venous thrombosis (CVT). Although the risk for PE is higher than in the general population (RR = 1.78 [95% CI 1.41-2.25]; P < 0.00001), in daily clinical practice it is considered a rare pulmonary manifestation in pSS patients, with an estimated frequency of less than 5% (16, 69, 70). The risk appears to be higher among hospitalized and recently discharged patients (16). Case reports of portal vein thrombosis or Budd-Chiari syndrome associated with pSS are anecdotal in the literature and were associated with antiphospholipid syndrome (APS) (71, 72). As with other autoimmune diseases, inflammation plays a critical role in the activation of the coagulation cascade (increasing tissue factor expression, downregulation of protein C or inhibition of fibrinolysis) and endothelial injury, while no specific comments can be made regarding venous stasis and chronic venous insufficiency (CVI), although both CVI and SS tend to occur in middle aged individuals (73). Hypercoagulability is also the result of anti-annexin antibodies, secondary APS, or medication (i.e. corticosteroids) (74). Although, little is known about the venous endothelial dysfunction in SS, it would be safe to assume that the same proinflammatory pathways and functionally abnormal lymphocytes described in the arterial endothelium dysfunction, will dysregulate the activity of the venous endothelium as well (75). Considering all these aspects, the risk for VT is not the same in all patients and the risk assessment process should be individualized. The patients should be advised to lower the impact of the general risk factors, such as smoking, low physical activity, body mass index, hormone replacement therapy. In the situation of surgery or malignancy a close surveillance of VT risk should be performed.

### 3.2 Cardiac Abnormalities

Cardiac involvement in pSS is less studied than in other rheumatic diseases, even though it appears that patients with pSS have a higher likelihood of heart failure than the general population (OR = 2.54 [95% CI 1.30-4.97]; P < 0.007) (16). During the last years, the development of more sensitive imaging techniques (i.e. myocardial deformation imaging by echocardiography and cardiac magnetic resonance imaging) brought us important evidence regarding the cardiac structural abnormalities present in patients with pSS, even in subclinical stages. However, clinically silent manifestations, such as left ventricular diastolic dysfunction have been described, possibly in the context of increased AS (76). Fatigue, one of the most common symptoms in patients with SS, is also a cardinal symptom in heart failure (HF), for which diagnosis can be troublesome at times in this context (77). Even asymptomatic, SS patients display a significantly higher prevalence of structural abnormalities, including valvular abnormalities, particularly mitral (29.90% vs. 10.71%, P < 0.001) and aortic regurgitation (23.36% vs. 9.82%, P = 0.007), pericardial effusion (8.41% vs. 0.89% P = 0.008), higher systolic pulmonary artery pressure and left ventricular diastolic dysfunction (78, 79). The latter seems to be more of concern, knowing that diastolic dysfunction is one of mechanisms leading to heart failure among patients with rheumatic diseases and that SS patients display significantly higher isovolumetric relaxation times and lower E wave deceleration times (52, 56, 76). Tissue Doppler echography (TDE) and speckle-tracking echocardiography are techniques able to detect subclinical myocardial alterations before ejection fraction changes. Regarding pSS, TDE revealed that both left ventricle systolic and diastolic functions are altered, with septal and lateral wall systolic myocardial wave velocities (Sm) being significantly lower in patients with pSS compared to healthy controls (7.5cm/sn ± 1.4cm/sn vs. 9.2cm/sn ± 1.6cm/sn, P < 0.001 and 7.9cm/sn ± 1.6cm/sn vs. 10cm/sn ± 2.4cm/sn, P < 0.001), as well as lower values for septal early diastolic myocardial wave velocities (Em) (8.4cm/sn ± 2.5cm/sn vs. 11.4cm/sn ± 2.6cm/sn, P < 0.001) and septal Em late diastolic myocardial (Am) wave velocities ratios (0.9 ±0.4 vs. 1.2±0.3, P < 0.02) (80). More advanced techniques, such as 4D- strain imaging confirmed that global longitudinal strain and global area strain are impaired in patients with pSS, while circumferential and radial strain are not, possibly because the longitudinally arranged fibers from the subendocardial layer are the first to be affected (81). As mentioned before, in the case of subclinical atherosclerosis, the disease duration plays a critical role, with a more evident left ventricle deterioration as the duration of the disease increased (81).

Multimodality imaging plays a pivotal role in the detection of myocardial involvement in systemic immune-mediated diseases (SIDs), enriching the information offered by echocardiography (5). Cardiac magnetic resonance (CMR) imaging with tissue characterization sequences (T1 and T2 weighted imaging, late gadolinium enhancement- LGE, and parametric mapping) and positron emission tomography (PET) provide additional insight regarding the presence of non-ischemic inflammatory myocardial involvement. Furthermore, the subepicardial or mid-myocardial LGE pattern is considered specific for myocardial injury associated with inflammatory conditions, differentiating from ischemic conditions (such as coronary artery disease) (5). In some diseases, such as rheumatoid arthritis and SSc, it has been shown to correlate with disease activity (82). A recent study involving asymptomatic SS patients revealed that myocardial fibrosis (identified by LGE on CMR) was independently associated with salivary gland focus scores higher of at least 3 (83, 84). Furthermore, the LGE-positive patients were found to have higher LV mass index and LV end-diastolic volumes compared to their LGE-negative peers, therefore suggesting that the stronger the lymphocytic infiltration into the salivary glands, the higher the chances for developing myocardial infiltration, edema and eventually fibrosis (84). CMR feature tracking (CMR-FT) is a relatively new and special post-processing technique used for assessing myocardial deformation, with ventricular strain being one of its applications. It was shown that patients with pSS without any cardiovascular disease, who associated Raynaud’s phenomenon, a focus score of at least 2 or an ESSDAI score of at least 8, have left ventricular regional dysfunction as shown by CMR-FT (85). Furthermore, a significant impairment in LV circumferential (P= 0.015) and longitudinal strain (P= 0.08), assessed by CMR-FT, was observed in patients with pSS, compared to healthy controls (85). CMR opened a new chapter for evaluating and diagnosing subclinical myocardial involvement, demonstrating so far that the heart is a vulnerable and critical target in SIDs, broadening the spectrum of known cardiac manifestations associated with SIDs (5). Its place in the management of patients with pSS is yet to be defined. However, current experience highlights an important prognostic value which will probably be confirmed by future studies.

Computed tomography (CT) is particularly valuable in pSS in assessing the pericardial involvement (pericarditis, pericardial thickening) and great vessels. The increased risk of aortic aneurysm and dissection observed among patients with SS warrants the importance of CT in the aortic disease diagnostic and surveillance (62).

**Figure 2:**
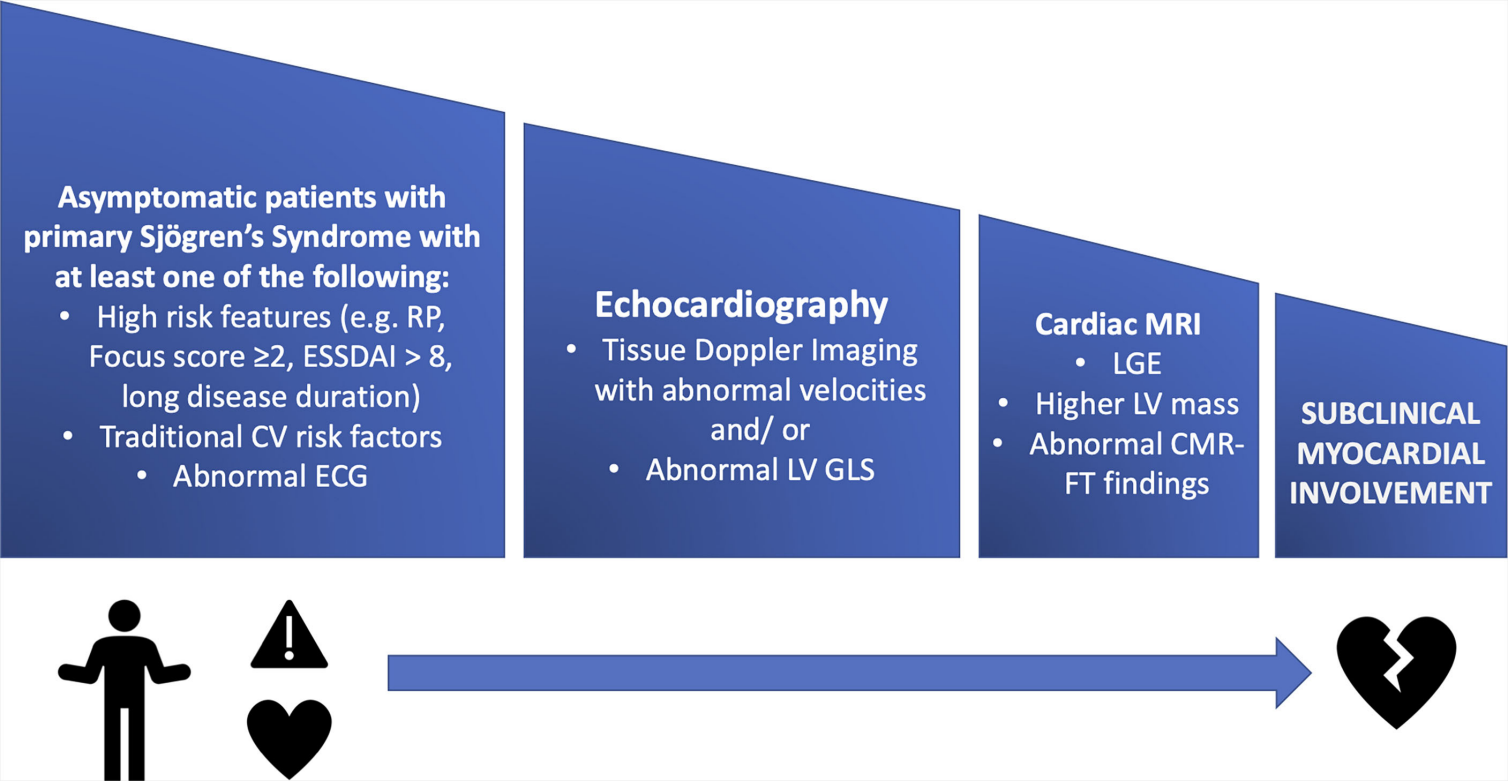
A stepwise approach for screening pSS patients for subclinical myocardial involvement using multimodality imaging. CMR- FT, cardiac magnetic resonance feature tracking; CV, cardiovascular; ESSDAI, EULAR Sjogren’s Syndrome Disease Activity Index; GLS, global longitudinal strain; LGE, late gadolinium enhancement; LV, left ventricle; MRI, magnetic resonance imaging; RP, Raynaud’s Phenomenon.

Cardiac imaging modalities should be performed in patients with symptoms or ECG abnormalities and according to current guidelines. Until now, the screening for structural cardiac manifestations is not recommended to all pSS patients (see **Figure 2**).

Infants and children with congenital heart block, born from anti-SSA/SSB antibodies positive mothers with SLE or pSS, are at risk of developing endocardial fibroelastosis (EFE), despite adequate pacing (86). EFE can develop early, in the first weeks after birth, or later, during childhood, presenting with progressive heart failure symptoms. Deposition of acellular fibrous and cartilaginous tissue in the subendothelial layer of the endocardium, more frequently involving the inflow tracts and apices of both ventricles leads to severe left ventricular diastolic impairment with restrictive cardiomyopathy, with a possible evolution towards dilated cardiomyopathy (87, 88). Other features are papillary muscles shortening with severe mitral regurgitation and mural thrombus formation (89, 90). CMR is also a valuable tool for diagnosing EFE in children born from anti-SSA/SSB antibodies positive mothers. Although the condition is rare, high mortality rates have been described in autoantibody-associated EFE, prompting the need for heart transplation (86). During the preconception consultation, this risk should be discussed with the family, as well as the need for long term monitoring of the infant.

### 3.3 Pulmonary Hypertension

Pulmonary hypertension (PH) was described in patients with SS and the mechanisms need to be carefully evaluated to have a correct therapeutical approach. PH secondary to lung disease is possible, as pulmonary involvement is not uncommon in SS, with small airways and interstitial lung diseases being the most frequent pulmonary manifestations associated with SS (69, 70). Left heart disease might be another possible cause of PH in patients with pSS. Overall, PH is encountered in less than 5% of patients affected by SS (70). Even so, a recent study revealed that patients with pSS have a higher incidence of hospitalisation related to PH, compared to the general population (aHR = 3.32) (91). Pulmonary arterial hypertension (PAH) is also considered a rare complication in patients with pSS (69). Conversely, the prevalence of pSS among patients undergoing initial evaluation for PAH may be higher than expected (92). RP is associated with higher values of pulmonary artery systolic pressure and is considered one of the predictors for PAH (59). Other features potentially associated with PAH in pSS are high titers of rheumatoid factor, pericardial effusion and hepatic injury (59). More recent findings suggest that PAH is more frequent in patients with a low index of disease activity, longer disease duration with early onset (although it could also be the initial symptom) and positivity for anti-SS-B and/or anti-U1-RNP antibodies (59, 93). Therefore, since PAH tends to occur in atypical, quiescent forms of disease, its real incidence could be underestimated, warranting periodical screening. Furthermore, compelling specific data regarding the use of vasodilators and immunosuppressive therapy in PAH associated with SS are lacking. Once diagnosed, and after careful exclusion of other mechanisms, PAH in patients with pSS should be managed with vasodilators according with existing international or national guidelines and with individualized immunosuppression.

It is known that obstructive sleep apnea (OSA) can induce pulmonary hypertension through hypoxia, as well as contributing to the cardiovascular risk of the individual. A recent study highlighted that hospitalized patients with pSS have a significantly higher incidence of OSA, independently of obesity (AHR= 1.97, [95% CI 1.70- 2.28]; P< 0.001) (91).

A particular and anecdotal report on pulmonary hypertension described in pSS is pulmonary veno-occlusive disease, which responded well to immunosuppressive therapy, without the need to use vasodilatators, even though data are very limited (94).

### 3.4 Autonomic Dysfunction

The autonomic nervous system deserves to be mentioned since it plays a key role in the regulation of the cardiovascular system and its dysfunction is prevalent among pSS patients, with a great impact on the quality of life (95). It appears that dysautonomia is the result of impairment in both parasympathetic and sympathetic nervous system, resulting in reduced heart rate and blood pressure variability (95). Reduced heart rate variability (HRV) is an independent predictor of a cardiac event and associated with sudden cardiac death in the general population (96). In a cohort of patients with pSS, autonomic dysfunction assessed by the HRV was observed in 35.7% of the cases, being associated with higher ESSPRI fatigue score (97). RP is also significantly more prevalent patients with pSS and autonomic dysfunction than in those without (29.4% vs. 14.4%, P = 0.048) (97). Orthostatic hypotension is another feature found in pSS with autonomic dysfunction, with symptoms ranging from postural lightheadedness to syncope (98). Clinicians should be aware about the impact of autonomic system dysfunction and its impact on the quality of life, as well as the potential impact on the risk of cardiac events, such as sudden cardiac death and syncope. Patients could benefit from additional tests (Holter ECG, tilt table test) and should be managed in an integrated approach, with the help of neurologists, cardiologists and rhemathologists.

### 3.5 Conduction Disease

Congenital heart block (CHB) may be associated with maternal antibodies against SS-A (Ro) or SS-B (La) proteins (neonatal lupus syndrome) (99). It generally occurs in 2% of the pregnancies of Ro-positive mothers, during the first 16-24 weeks of gestation, with high recurrence rates for subsequent pregnancies (100). The mortality rates in infants affected by CHB are relatively high (up to 30%) and most live-born children will require permanent pacing, with data indicating that pacing should be done as early as possible, considering symptoms, QRS escape rhythm width, left ventricular dysfunction or mean ventricular rate (101, 102). Anti-Ro52 antibodies display a high affinity for fetal cardiac cells, inducing apoptosis as they initially bind to them. This translates into a first-degree atrio-ventricular (AV) block, which can be transient. However, as anti-Ro and anti-La antibodies continue to bind to the apoptotic cells, macrophages will start clearing them, leading to an inflammatory reaction and permanent damage to the AV node with second or third-degree AV block (101). It should be mentioned that even though anti-SSA-A/SS-B antibodies play a key pathogenic role in CHB, there are also additional risk factors (maternal MHC genes, maternal age, maternal interferon signature, maternal positivity for both anti-Ro52 and anti-Ro60 antibodies, high titers of anti-p200 antibodies, fetal MHC genes, low levels of vitamin D, infections), considering that CHB occurs in only a minority of pregnancies from SS-A-positive mothers (100, 101). HCQ is currently the only pharmacological prophylaxis indicated for CHB and is considered safe to use in pregnancy, as well as breastfeeding. Notably, HCQ reduces by half the recurrence rates if used in a 400 mg daily dose from the 10^th^ week of gestation in mothers who gave birth to and infant with CHB (100). Fetal heart monitoring by echography remains the gold standard of evaluation, with PR interval prolongation assessed by echography being an indispensable tool for the timely diagnosis of fetal cardiac tissue injury and initiation of steroid maternal treatment.

### 3.6 Electrical Abnormalities in pSS

The QT interval reflects the duration of the ventricular action potential, which can be further divided into depolarization and repolarization. Higher degrees of dispersion in ventricular repolarization will result in longer QT intervals. On this basis, QT prolongation is one of the most clinically relevant ECG findings, being associated with potentially life-threatening ventricular arrhythmias (such as torsade de pointes), syncope and even sudden cardiac death. The issue of acquired long-QT syndromes (LQTS) in SS and similar diseases has been described from various perspectives. In order to better address the LQTS, a synthetic approach, based on the implications of the disease itself and of the treatment options currently in use, will be used. QT prolongation can occur in patients with SS as a consequence of inflammation (not specific to SS) and circulating anti-SS-A antibodies (103, 104). A significant relationship between the degree of inflammation, assessed by CRP levels or soluble TNF-receptor-1 levels, and QT duration has been observed in the general population (103, 105, 106). In addition, CRP levels and IL-6 levels were found to predict sudden cardiac death even in the general population, at least partly explained by a higher propensity to develop long-QT associated arrhythmias, while among patients with RA treated with the IL-6 receptor antibody tocilizumab, a significant shortening of the QT interval was observed (107–109). Furthermore, anti-SS-A/SS-B antibodies can interact with potassium channels, directly affecting the ventricular repolarization and the QT interval, much like an autoimmune cardiac channelopathy (110). Autoimmune long-QT syndromes also generate a higher incidence of ventricular arrhythmias and sudden cardiac death (110). Concerns regarding the use of hydroxychloroquine (HCQ), one of the most used drugs in SS, and cardiac arrhythmia have been based on the fact that HCQ can prolong the QT interval. However, in a recent ample study, it was shown that HCQ did not increase the risk of ventricular arrhythmia (regardless of cumulative dose or duration of treatment) in patients with various autoimmune diseases, including SS (111). The role of the ECG-based screening for the detection of LQTS in patients with SS is not clear. However, patients should have a baseline ECG at diagnostic and probably yearly ECG during follow-up. The clinical situations that increase QT interval (i.e. dyselectrolitemia, drugs) should be avoided. Patients with LQTS who are symptomatic (i. e. palpitations, syncope), should undergo Holter monitoring in order to assess the arrhythmic burden or the existence of high-risk arrhythmic events, such as ventricular tachycardia.

## 4 Current Evidence on Cardiovascular Prevention

Primary prevention is the cornerstone intervention in populations with a higher than normal risk for developing cardiovascular diseases. Even if this particular population is not yet formally considered a high risk one in terms of cardiovascular diseases by the current prevention tools, clinicians should be aware that patients with pSS have a higher risk for developing cardiovascular diseases, as previously discussed (16, 17). Furthermore, these patients have a higher prevalence of cardiovascular risk factors, such as hypertension, hypertriglyceridaemia, OSA and possibly diabetes mellitus (15–20, 91). We should mention that no specific recommendations have been formulated in the primary prevention of patients with pSS (30). In the meantime, it is advisable to use the prevention tools as for the general population and to thoroughly assess and address the traditional cardiovascular risk factors (30). In terms of secondary prevention, patients should adhere to recommendations and thresholds recommended by the specific guidelines.

## 5 Major Gaps and Limitations

One of the major limitations encountered is that although there are many published reviews and studies concerning the cardiovascular manifestations in autoimmune diseases, it is unclear to what extent we can extrapolate this knowledge to SS, as data specifically addressing this disease is limited, at least when compared to RA or SLE. Furthermore, it would seem factitious to do so considering that SS is not the typical inflammatory rheumatoid disease and that the inflammatory pathways involved in RA for instance are different than the ones described in SS. Accordingly, pSS patients rarely require immunosuppressive therapies since systemic inflammation, at least quantified by traditional markers is usually absent or mild. Therefore, it becomes clear that different proinflammatory pathways are involved in pSS and their complex interaction with the cardiovascular system should be further studied.

## 6 Conclusion

Although to an extent patients with pSS share the same cardiovascular risk profile as the general population, the superimposed disease specific risk factors can dramatically alter their prognosis, even if current cardiovascular risk scores do not acknowledge pSS as a comorbidity. Therefore, we propose an individual-based approach taking into account cardiovascular and disease-specific risk factors, and using modern imaging techniques for subclinical myocardial and vascular involvement screening. Further studies should attempt to address these issues specifically in pSS and compare the results to other autoimmune diseases (RA or SLE) in which the issue of cardiovascular diseases is far more established.

## Author Contributions

MC and RJ contributed to the conception and design of the article. MC and AD researched the literature for relevant articles/studies. MC, AD and AM drafted the article. CJ, SS and RJ revised it critically for important intellectual content. MC, AM and RJ designed the figures. CJ and RJ designed the table. All authors agreed on the final form. All authors contributed to the article and approved the submitted version.

## Funding

This review was supported by University of Medicine and Pharmacy "Carol Davila", Bucharest, Romania.

## Conflict of Interest

The authors declare that the research was conducted in the absence of any commercial or financial relationships that could be construed as a potential conflict of interest.

## Publisher’s Note

All claims expressed in this article are solely those of the authors and do not necessarily represent those of their affiliated organizations, or those of the publisher, the editors and the reviewers. Any product that may be evaluated in this article, or claim that may be made by its manufacturer, is not guaranteed or endorsed by the publisher.
